# Cancer survivorship, excess body fatness and weight-loss intervention—where are we in 2020?

**DOI:** 10.1038/s41416-020-01155-2

**Published:** 2020-11-25

**Authors:** Annie S. Anderson, Richard M. Martin, Andrew G. Renehan, Janet Cade, Ellen R. Copson, Amanda J. Cross, Chloe Grimmett, Laura Keaver, Angela King, Elio Riboli, Clare Shaw, John M. Saxton, On behalf of the UK NIHR Cancer and Nutrition Collaboration (Population Health Stream), Annie Anderson, Rebecca Beeken, Janet Cade, Amanda Cross, Angela King, Richard Martin, Giota Mitrou, Elio Riboli, John Saxton, Andrew Renehan

**Affiliations:** 1 Centre for Research into Cancer Prevention and Screening, Division of Population Health & Genomics, University of Dundee Ninewells Hospital and Medical School, Dundee, DD1 9SY UK; 2 Bristol Medical School: Population Health Sciences, University of Bristol, Canynge Hall, Bristol, BS8 2PS UK; 3 MRC Integrative Epidemiology Unit, University of Bristol, Oakfield House, Bristol, BS8 2BN UK; 4 University Hospitals Bristol NHS Foundation Trust National Institute for Health Research Bristol Biomedical Research Centre, University of Bristol, Bristol, UK; 5 The Christie NHS Foundation Trust, Manchester Cancer Research Centre, NIHR Manchester Biomedical Research Centre, Division of Cancer Sciences, School of Medical Sciences, Faculty of Biology, Medicine and Health, University of Manchester, Wilmslow Road, Manchester, M20 4BX UK; 6 Nutritional Epidemiology Group, School of Food Science and Nutrition, G11, Stead House, University of Leeds, Leeds, LS2 9JT UK; 7 Wessex Genomic Medicine Centre, Cancer Sciences Academic Unit, University of Southampton, Southampton General Hospital, Tremona Road, Southampton, SO16 6YD UK; 8 Department of Epidemiology and Biostatistics, School of Public Health, Imperial College London, Norfolk Place, London, W2 1PG UK; 9 School of Health Sciences, University of Southampton, Southampton, SO17 1BJ UK; 10 Department of Health and Nutritional Science, Institute of Technology Sligo, Sligo, F91 YW50 Ireland; 11 NIHR Cancer and Nutrition Collaboration, Level E and Pathology Block (mailpoint 123), Southampton General Hospital, Tremona Road, Southampton, SO 16 6YD UK; 12 Biomedical Research Centre at The Royal Marsden and the Institute of Cancer Research, Fulham Road, London, SW3 6JJ UK; 13 Department of Sport, Exercise & Rehabilitation, Faculty of Health and Life Sciences, Northumbria University, Northumberland Building, Newcastle upon Tyne, NE1 8ST UK; 14 Public Health Nutrition, Centre for Research into Cancer Prevention and Screening, University of Dundee, Dundee, UK; 15 Yorkshire Cancer Research University Academic Fellow, University of Leeds, Leeds, UK; 16 Nutritional Epidemiology and Public Health, University of Leeds, Leeds, UK; 17 Cancer Epidemiology, Imperial College London, London, UK; 18 Public Representative, NIHR Cancer and Nutrition Collaboration, Southampton, UK; 19 Clinical Epidemiology, University of Bristol, Bristol, BRC UK; 20 Research Funding & Science External Relations, WCRF, London, UK; 21 Cancer Epidemiology and Prevention, Imperial College London, London, UK; 22 Clinical Exercise Physiology, Northumbria University, Newcastle, UK; 23 Cancer Studies and Surgery, University of Manchester; Honorary Consultant Colorectal Surgeon, Manchester, UK

**Keywords:** Risk factors, Cancer prevention, Weight management

## Abstract

Earlier diagnosis and more effective treatments mean that the estimated number of cancer survivors in the United Kingdom is expected to reach 4 million by 2030. However, there is an increasing realisation that excess body fatness (EBF) is likely to influence the quality of cancer survivorship and disease-free survival. For decades, the discussion of weight management in patients with cancer has been dominated by concerns about unintentional weight loss, low body weight and interventions to increase weight, often re-enforced by the existence of the obesity paradox, which indicates that high body weight is associated with survival benefits for some types of cancer. However, observational evidence provides strong grounds for testing the hypothesis that interventions for promoting intentional loss of body fat and maintaining skeletal muscle in overweight and obese cancer survivors would bring important health benefits in terms of survival outcomes and long-term impact on treatment-related side effects. In this paper, we outline the need for studies to improve our understanding of the health benefits of weight-loss interventions, such as hypocaloric healthy-eating plans combined with physical activity. In particular, complex intervention trials that are pragmatically designed are urgently needed to develop effective, clinically practical, evidence-based strategies for reducing EBF and optimising body composition in people living with and beyond common cancers.

## Background

Improvements in the early detection and treatment of cancer have led to a dramatic increase in the number of cancer survivors—those people alive who have been diagnosed with cancer before, during and after treatment.^1^ Globally, public health surveillance data show that 5‐year net survival rates from colon, rectal and breast cancers have increased steadily in the majority of developed countries,^2^ and, in the United Kingdom, the number of cancer survivors is expected to reach 4 million by 2030.^3^ The definition of survivors includes individuals who have been cured by treatments or who are on the road to recovery and aiming to reduce the risk of recurrence, as well as those living with metastatic disease, for whom efforts are more focussed on maximising treatment effectiveness, managing the side effects of treatment and preserving quality of life.

As we celebrate extended cancer survivorship, however, we must also be mindful of the co-morbid conditions,^4^ including overweight and obesity characterised by excess body fatness (EBF) that can affect the quality of those additional years. Body mass index (BMI) is the measure most commonly used as a proxy for EBF; the measure becomes notable when the value increases beyond 25 kg/m^2^ (overweight) and is deemed substantial at levels above 30 kg/m^2^ (obesity). It is estimated that, worldwide, 1.9 billion adults and over 340 million children and adolescents are now living with overweight or obesity.^5^ Although EBF has been identified as a risk factor for at least 13 different types of cancer,^6^ its effect on cancer survivorship is less clear. However, the prevalence of EBF in Western societies means that its probable influence on the quality of cancer survivorship and the prospect of prolonged disease-free survival after primary curative treatment cannot be ignored. The effects of EBF on insulin resistance, systemic inflammation and other circulating factors such as adipokines and sex hormones, which are linked to primary cancer risk, are well-described,^6^ and research into the biological mechanisms that underlie the obesity–cancer relationship (both in tumour initiation and progression) is ongoing.^7^ EBF can influence the quantity, distribution and quality of adipose tissue, which is now recognised to comprise not just adipocytes, but also blood vessel stromal cells and immune cells. Accordingly, the roles of adipose tissue have been found to extend beyond triacylglycerol storage to include (among many others) glucose and lipid metabolism, appetite regulation and, notably, immunity and inflammation, providing potential mechanisms by which EBF might influence cancer survivorship and response to treatment, as well as risk.^8,9^

Several leading health authorities recommend the management of excess weight (e.g., avoiding weight gain, intentional weight loss and weight-loss maintenance) for people living with and beyond cancer,^10–12^ but service provision and resources for health behaviour change and the promotion of effective interventions within healthcare systems is suboptimal.^13^ Weight management in cancer patients has routinely been dominated by concerns about unintentional weight loss (secondary to cancer treatments or due to progressive disease) and low body weight. These concerns have resulted in an emphasis on nutritional interventions to maintain or increase weight because of the negative outcomes associated with loss of body mass in people with advanced cancer. Nutrition- screening tools focus on parameters of undernutrition with little heed to the issues and adverse risk profile of patients who have EBF at diagnosis, or who gain further weight (body fat) during treatment and beyond.

Consideration of the health benefits of managing EBF is largely overlooked. There is a perception that many clinicians fail to be convinced that interventions related to EBF are a key part of cancer care and will be beneficial to patient outcomes.^13^ Clinicians might even avoid these issues because they are concerned about evoking feelings of guilt or undermining patient–health-professional relationships (especially where BMI is a known risk factor for the cancer site),^13,14^ despite opportunities (‘teachable moments’) to address this issue during and after cancer treatment.

The influence of intentional weight loss on adipose tissue biology is unknown. It is possible that some effects of obesity might be imprinted, and therefore might not be reversible.^7^ On the other hand, work in mouse models suggests that intentional weight loss through caloric restriction boosts anticancer immune surveillance and delays progression.^8^ It is also possible that these biological responses could enhance treatment outcomes and risk of disease recurrence. The importance of understanding more about the impact of obesity on both cancer incidence and outcomes was identified in 2020 as one of the eight research-priority areas needed to accelerate progress in cancer management by the American Society for Clinical Oncology.^15^ In this paper, we outline the need for intervention trials to address the issue of whether promoting intentional loss of body fat and maintaining skeletal muscle in overweight and obese cancer survivors would bring important health benefits in terms of survival outcomes and long-term impact on treatment-related side effects. Realistically, management of EBF is unlikely to become a core part of survivorship plans, unless robust clinical trials and subsequent clinical guidelines can be developed.

## EBF and cancer survival

Growing evidence from epidemiology studies indicates that avoiding EBF might have a role in reducing cancer morbidity and mortality worldwide. The Global Burden of Disease Study reported (using various ecological assumptions) in 2019 that amongst 896,040 colorectal cancer deaths occurring in 2017, 73,475 (8.2%) were attributable to a high BMI.^16^ A meta-analysis of 82 studies reported a 35% increase in breast-cancer-related mortality and a 41% increase in all-cause mortality in women with breast cancer who were obese, independent of menopausal status.^17^ Similarly, meta-analyses suggest that obesity is associated with poorer survival outcomes in bladder,^18^ prostate^19^ and hepatocellular^20^ cancer patients.

### The obesity paradox

**Fig. 1:**
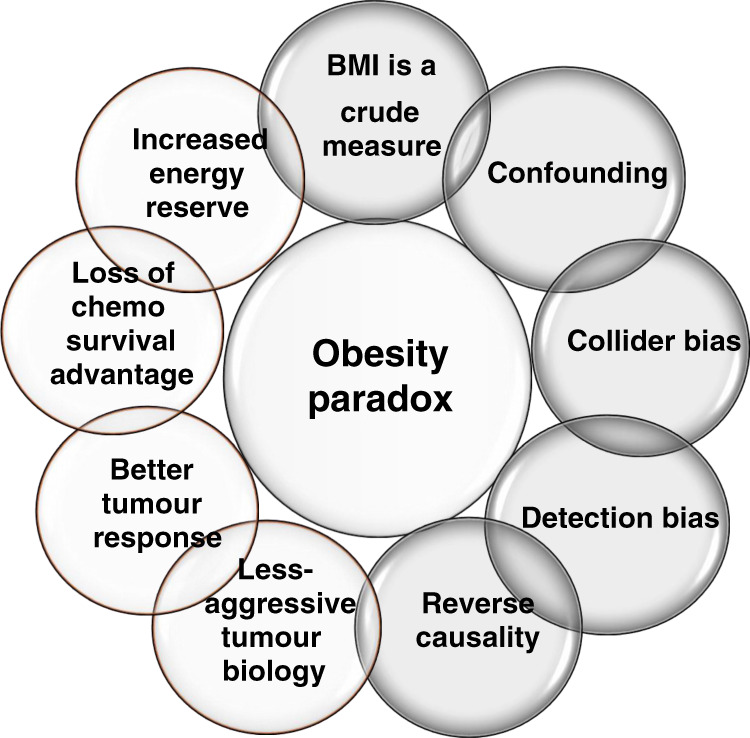
Possible explanations for the obesity paradox. Despite significant evidence that excess body fat (EBF) is associated with reduced cancer survival, data from a number of studies indicate that overweight and early obese cancer patients exhibit improved survival—this is known as the so-called ‘obesity paradox’. Although there are potential clinical and biological explanations for this in specific patient groups many of these reports can be explained by methodological mechanisms, including the inadequacy of BMI as a measure of adiposity.

Considerable debate surrounds the ‘obesity paradox’,^21,22^ in which high body weight appears to be associated with survival benefits after diagnosis of colorectal,^23^ endometrial^24^ and lung cancer.^25^ In some studies, this phenomenon can be explained by the association of obesity with less aggressive tumour subtypes, such as the increased incidence of type 1 tumours, which have a good prognosis, compared with type 2 tumours, which have poor prognosis, in obese endometrial cancer patients.^26^ A higher tolerance of some systemic anticancer therapies in overweight/obese patients and the benefit of energy reserves to support the body during the stress of anticancer therapies have also been postulated as clinical explanations for the obesity paradox (Fig. 1). In some cases, higher body weight might reflect greater fat-free mass that may increase the responsiveness to treatment regimens.^27^ However, in many publications, the association of enhanced survival with overweight or obese status is an artefact of methodological issues. These issues commonly include combining cohorts of patients with early and advanced cancer so that observational data are confounded by disease-related weight loss (reverse causality) and the use of heterogenous cohorts that fail to adjust for tumour biology, stage or treatment or other confounders such as smoking. Other reported causes of the obesity paradox outlined in Fig. 1 include detection bias, where patients undergoing medical investigation for obesity-related co-morbidities are diagnosed with incidental early-stage cancers, and collider bias, a specific form of selection bias demonstrated in the relationships between smoking, cancer and obesity. Cancer patients who are not obese might have other risk factors, such as smoking, and an inverse association is therefore artificially strengthened between obesity and cancer outcomes. Longer-term cohort studies that have the potential to provide better repeated measures over time are needed. Finally, assessment of obesity by BMI fails to take body composition, notably body fat distribution, into account. At the most basic measurement, this would include markers of central obesity such as waist circumference.

### Weight gain after diagnosis and survival outcomes

Data regarding weight gain after diagnosis of common cancers add another layer of complexity to the link between EBF and cancer morbidity and mortality. For example, whereas poorer survival outcomes associated with weight gain are suggested for breast cancer after diagnosis,^28^ current evidence for the influence of weight gain after diagnosis on colorectal cancer survival seems to be less clear-cut,^29^ notably when patients with early disease and those with metastatic disease (and high tumour burden) are included in the same analysis. Although some studies suggest that a higher BMI might be associated with better survival in patients with colorectal cancer, meta-analyses have reported little impact on the risk of survivorship in overweight patients, whereas both obese and underweight patients have an increased risk of all-cause mortality, cancer-specific mortality, disease recurrence and worse disease-free survival compared with patients of normal weight.^30^ Being able to distinguish between intentional and unintentional weight loss is also important, as is the impact of weight loss on body composition—specifically, a reduction in EBF while maintaining lean body mass is desirable. In addition, certain treatment modalities are associated with weight gain, including endocrine therapy in breast and prostate cancer, and steroid treatments used as an adjunct to many chemotherapy regimens and as supportive care in many oncological emergencies associated with advanced cancer. These factors highlight the importance of investigating EBF and weight gain by treatment. Added to this, methodology concerns, including sampling selection bias, residual or unmeasured confounding factors, reverse causation and collider bias, call into question the epidemiological basis for the obesity paradox in this context.

### EBF, skeletal muscle mass, surrogate measures and survivorship

A growing number of observational studies have relied on surrogate measures of adiposity (e.g., body weight, BMI and waist circumference), which do little to advance our understanding of how changes in the key body composition parameters of EBF and skeletal muscle mass might independently influence cancer survivorship. Caan et al.^31^ argued that people who are overweight or obese generally have higher levels of skeletal muscle than people of lower weight, thus decreasing the risk of disease recurrence, surgical complications and treatment-related toxicities associated with lower skeletal muscle mass. It is, however, important to analyse appropriately for age when classifying sarcopenia.^32^ When age is taken into consideration, sarcopenic obesity— skeletal muscle depletion despite high BMI—is reported to be prevalent in approximately one-tenth of patients with advanced solid tumours, and is independently associated with increased complication and mortality rates across multiple cancer sites and treatment plans.^33^ Furthermore, in non-metastatic breast-cancer patients, computer-tomography-derived measures of sarcopenia and total adiposity at diagnosis were shown to be independently associated with overall mortality over 6 years of follow-up, whereas BMI was not.^34^ These results further underline the need to assess body composition rather than rely on BMI in order to guide best advice for nutritional and physical activity survivorship plans.

## The effect of EBF on cancer treatment

The effects of EBF and weight-management interventions on treatment outcomes, post-treatment morbidity and mortality might differ between cancer types, and many important research questions in this arena need to be answered.^35^ For example, the impact of high BMI (reflecting EBF) on the efficacy of local and systemic cancer therapies and the associated side effects in the context of optimising long-term treatment plans is largely understudied.^36^ A systematic review of the effect of obesity on toxicity in women treated with chemotherapy in early-stage breast cancer concluded that obese patients tolerate chemotherapy better than lean patients.^37^ However, it was acknowledged by the authors that this observation ‘may be confounded by poorly specified dose-capping practices and the use of haematopoietic growth factors' (which may have been used more frequently in obese patients if clinicians perceived that these patients were at a higher risk of myelosuppression due to higher absolute drug doses).

A narrative review^34^ evaluating the effect of obesity on a wide variety of oncology-treatment modalities highlighted a number of points. First, technical challenges posed by high BMI might adversely impact surgical morbidity outcomes (e.g., increased risk of surgical site infections, reduced lymph-node harvest and increased risk of margin positivity). Second, the potential exists for suboptimal chemotherapy dosing; this is associated with capping chemotherapy in obese patients to avoid toxicity and might be a driver of poor prognostic outcomes. Conversely, however, the efficacy of immune-checkpoint inhibition could potentially be enhanced in patients who are obese. These checkpoints moderate the immune response and the ability to impact on tumour cells. Immunotherapy agents have been developed for a number of cancers and the importance of these in the overweight and obese is emerging.^34^

The review also raised an important question: does EBF influence outcomes directly through cancer biology (such as via the effects of adipose tissue on the levels of oestrogens, insulin, insulin-like growth factors and other adipokines to create a pro-inflammatory environment that encourages carcinogenesis) or are the adverse outcomes of EBF mediated through indirect pathways (e.g., chemotherapy dosing) that result in suboptimal treatment?

### Interpreting the results of observational studies investigating the effect of EBF on mortality and survival

Various studies have investigated the impact of EBF on a range of cancer outcomes in many cancer types but, to date, the evidence on overall survivorship risks is inconclusive. In summarising these studies (e.g., in breast cancer), the World Cancer Research Fund (WCRF) Continuous Update Project (CUP) panel^10^ developed a framework for interpreting the effect of anthropometric measures on mortality and survival at three key time points: pre-diagnosis of cancer, peri-diagnosis/peri-treatment, and during survivorship (see Table 1). Exposures (diet, physical activity and body composition) measured prior to cancer diagnosis are anticipated to influence cancer incidence and overall mortality via an effect on cancer biology. The main biological mechanisms of interest (metabolic regulators including insulin, insulin-like growth factor 1, adipokines, inflammation-related molecules and steroid hormones, as well as the cellular and structural components of the tumour microenvironment, including adipose tissue)^38^ are likely to have long-term impacts without appropriate interventions.

**Table 1:** Interpretation of studies evaluating anthropometric measures on mortality and survival.

When measure determined	Time zero in modelling	Endpoint terminology	Interpretation
Pre-diagnosis	At cohort entry	Cancer mortality	Cancer mortality and all-cause mortality among cohort participants is conditional on the exposure influencing cancer incidence, subsequent treatment and cancer biology. These studies indicate the burden of death attributed to anthropometric exposures (e.g., EBF). The findings have implications for public health and global policy, but do not have direct implications for weight- management intervention strategies in cancer survivors.
Peri-diagnosis/peri-treatment	At diagnosis or start of treatment	Survival	These studies are best considered as prognostic studies and should be interpreted in light of adjustment for other major cancer prognostic factors, including disease stage, treatment, and performance status. There is often a proportion of patients who have lost weight due to cancer and treatment (reverse causality), with downward BMI-category migration. These studies add some evidence to directly inform weight- management policies in cancer survivors.
Post treatment	During survivorship, e.g., at year 1	Survival	These studies in patients have already survived treatment. Survival endpoints are important but so are quality of life, late effects of cancer treatment, avoidance of other cancer events and other diseases. These studies add evidence to directly inform weight- management policies in cancer survivors.

Interventions based on these exposures are thus relevant to cancer-prevention strategies, but further evidence will be required for weight-management policies in cancer survivors. Anthropometric measurements taken at the time of cancer diagnosis can be assessed as prognostic indicators, but must be interpreted in the context of the cancer type, stage and patient performance status, as well as the timing of measurements in relation to treatment modalities. The impact of body composition on therapy-related toxicities is equally important in patients with advanced cancer where the goals of systemic therapies are to improve and maintain quality of life whilst also extending life expectancy. This area is poorly addressed in the current literature and represents an important unmet research need. However, as recent weight loss is a frequent presentation of advanced-stage cancer (reverse causality), there is a need to analyse the association of body mass and survival in advanced-stage patients separately to that of patients with early-stage disease who are less likely to present with weight loss and will have a longer median survival time.

Assessment of body mass and size after treatment also needs attention in relation to the type of treatment received for different tumour types and any associated toxicities, and an awareness of selection against patients with rapid disease progression who have not survived to this point.

### Weight-loss trials: a gap in the evidence

Despite the limitations of observational data, the consistency and magnitude of associations between EBF/weight gain and survival outcomes for some cancers reported in systematic reviews and meta-analyses support the need for intervention studies.^27,39^ To date, weight-loss intervention studies have predominantly been carried out in breast-cancer survivors. A large-scale dietary intervention trial (low fat, high fruits and vegetables) in women with early-stage breast cancer—the Women’s Intervention Nutrition Study (WINS)—was successful in supporting women to lose weight, with indications of lower cancer recurrence in the intervention group, notably in women with oestrogen-receptor (ER)-negative disease.^40^ Furthermore, a growing number of short-term trials have demonstrated the effects of intentional weight loss on blood-borne biomarkers of cancer and cardiometabolic risk, including changes in serum sex hormones,^41^ inflammation markers^42^ and insulin sensitivity.^43^ A small number of ongoing intentional weight-loss trials are also ongoing in breast-cancer survivors^44–46^ and are expected to report on survival and associated outcomes over the next decade. Weight-loss trials have also been undertaken in endometrial cancer survivors.^47^ However, a 2018 Cochrane review^48^ concluded that there is insufficient high-quality evidence to determine the effect of interventions on survival, quality of life or cardiovascular events. The authors highlighted problems of high risk of bias by failing to blind personnel and outcome assessors, and significant losses to follow-up. They also emphasised the need for adequately powered trials with a follow-up of at least 5–10-year duration.

Importantly, no trial has yet established the effect of intentional weight loss following a cancer diagnosis on mortality, and many gaps in our understanding of how to optimise such interventions remain. The optimal contributions of diet composition, caloric intake, amount and nature of physical activity (including sedentary time) for promoting loss of EBF and avoiding weight gain^49^ are important considerations for future intervention research. Furthermore, the effects of weight- management interventions on treatment-related side effects, as well as bone health, physical function, psychosocial issues and quality of life, have not been clearly defined for many cancers, and intervention studies are needed to address these important issues.^48^

Weight-management strategies in overweight and obese cancer survivors might also have a role to play in the prevention of non-cancer deaths—for some individual patients, the presence of EBF might also confer a poorer prognosis for survival from non-cancer disease. For example, cancer patients who also have diabetes have a decreased overall survival compared with cancer patients without diabetes, in part because they are at increased risk of non-cancer (mainly cardiovascular) deaths,^50^ which might be further increased by certain treatments (e.g., anthracycline chemotherapy).

Whilst the case for examining the impact of weight management can be made from current evidence, the design of programmes to capture the magnitude of effect and possible negative consequences need to be fully explored.

## Time to invest in intervention research for EBF?

Developing and testing interventions for promoting the intentional loss of EBF and maintaining skeletal muscle mass require a number of considerations, which we outline below.

### Optimum timing of interventions

The optimum window for weight-loss interventions in cancer survivors needs careful consideration. Treatment for cancer is increasingly being delivered over longer periods of time and is multimodal in nature; acute side effects, including unintentional gains in body weight and changes in body composition, which might negatively influence cancer outcomes and response to treatment, are not uncommon.^51^ Of early-stage breast-cancer patients receiving chemotherapy, 30–60% gain significant weight. This weight gain involves losing skeletal muscle while gaining adiposity^52^ and adversely impacts quality of life and overall health.^53^ Young breast-cancer patients can gain over 5% body weight in the first 12 months after diagnosis,^54^ which is associated with changes in eating habits resulting from emotional stress as well as the side effects of treatments (e.g., steroids and chemotherapy-induced menopause, cancer-related fatigue and reduced physical activity). Clearly, interventions that provide the support needed to help patients avoid or limit unintentional weight gain during treatment and/or facilitate EBF loss following completion of treatment whilst maintaining adequate levels of physical activity would be valuable adjuncts to curative cancer-care pathways.

Changes in nutritional and metabolic status that influence sarcopenia and cachexia must be addressed with the appropriate nutritional support throughout treatment,^11^ irrespective of body weight. For this reason, intentional weight-loss interventions might be challenging and possibly inadvisable for some cancer populations during the period of treatment, and the post-treatment period is likely to offer a more practical time frame. For example, chemoradiation treatment for patients with head and neck cancers is already associated with a significant incidence of weight loss and malnutrition, and patients frequently require nutritional support during treatment, while patients with upper gastrointestinal cancer often present with rapid weight loss owing to dysphagia and, again, management should be focussed on optimising nutritional intake prior to and during treatment.

### The study population

Careful consideration needs to be given to the study population, including age, location, ethnicity, co-morbidities, primary cancer site and stage of disease when designing weight-loss interventions aimed at optimising efficacy and effectiveness. Trials to investigate the benefits of intentional weight loss are most likely to be acceptable to clinicians and patients in cancer populations where there is evidence that EBF is associated with second cancer risk or poorer outcome. In addition, low frequency of rapid weight loss at presentation or associated with common first-line treatment strategies will also make intentional weight-loss programmes seem more appropriate. Patients with early-stage presentations of breast, endometrial, colorectal and prostate cancers might meet these requirements. Close attention must also be paid to the biology of the disease, particularly within metastatic cancer populations: patients with ER-positive metastatic breast cancer and no visceral disease frequently have an indolent disease course that can be managed predominantly by endocrine therapy over many years and constitute, potentially, a more appropriate population for weight-intervention strategies than patients with triple-negative metastatic disease who frequently develop rapid disease progression, leading to failure of vital organs.

### Outcome measures

Outcome measures in weight-management trials should include those that are patient-reported as well as clinically reported. Patient-reported outcomes (PROMS) include measures of quality of life, which can be broadly categorised into five groups: general health and well-being, physical factors (e.g., weight loss), symptoms (e.g., pain, nausea and fatigue), psychological factors (e.g., anxiety, insomnia and self-esteem) and social factors (e.g., relationships and work). Clinical outcome measures might vary according to cancer site and treatment regimens, but should include those assessing acute and long-term side effects of local and systemic therapies (e.g., lymphoedema volumes, fatigue scores, bone mineral density and cardiac ejection fractions) as well as cancer outcomes (locoregional and distant disease-free survival) and overall survival. Circulating biomarkers and surrogate endpoints (e.g., adenomas, breast density^55^) should be used alongside PROMS to gain an overview of the relevant biological and well-being perspectives allowing clinical, scientific and person-specific characteristic insights into the impact of interventions.

### Minimising heterogeneity/standardising outcomes

The sources of heterogeneity need to be carefully considered and controlled for in the design of weight-management studies, and/or considered during the analytic phase. The potential for clinical heterogeneity in outcomes exists according to disease subtype, stage and grade, as well as in the treatment received, but methodological heterogeneity in the way outcomes are defined can also occur. It is plausible that patients with different cancers might respond differently to weight- management interventions—notably, those with obesity-related cancers versus non-obesity-related cancers. Standardising outcomes is important for consistency and for comparison across trials, and allows incorporation into meaningful meta-analyses. To improve the definition and measurement of outcomes, the Core Outcome Measures in Effectiveness Trials (COMET) initiative^56^ provides guidance for researchers by advocating a standardised set of outcomes that should be measured and reported, as a minimum, in all clinical trials of health, including weight management. Examples listed in Table 2 illustrate the breadth of outcomes, similarities and differences by site used by different research teams, and highlight the need for further work on agreed core outcomes. Additionally, incorporation of the accumulating data to optimally predict obesity treatment (ADOPT)^57^ biological domain framework could advance the understanding of individual variability in response to adult obesity treatments and explore the physiological mechanisms that could influence cancer recurrence.

**Table 2:** Range of core outcomes relevant in clinical trials of weight management.

Cancer type	Specific clinical setting	No. of core measures	Description of core outcome sets
Breast cancer^69^	All treatments	27	**Survival and disease control:** Overall survival, death attributed to breast cancer and recurrence-free survival **Degree of health:** Overall well-being, physical functioning, emotional functioning, cognitive functioning, social functioning, ability to work, anxiety, depression, insomnia, financial impact, pain, fatigue, sexual function and body image **Patients treated with surgery and/or radiotherapy:** Satisfaction with breast(s), arm symptoms, breast symptoms and lymphoedema **Patients with systemic therapy:** Vasomotor symptoms, peripheral neuropathy, vaginal symptoms and arthralgia **Disutility of care:** Reoperation owing to involved margins, severity of acute complications (based on the assessment of postoperative outcome scores)
Prostate cancer^70^	Localised	19	12 apply to **all interventions**: Death from prostate cancer, death from any cause, local disease recurrence, distant disease recurrence/metastases, disease progression, need for salvage therapy, overall quality of life, stress urinary incontinence, urinary function, bowel function, faecal incontinence and sexual function Seven were **intervention-specific**: Perioperative deaths (surgery), positive surgical margin (surgery), thromboembolic disease (surgery), bothersome or symptomatic urethral or anastomotic stricture (surgery), need for curative treatment (active surveillance), treatment failure (ablative therapy) and side effects of hormonal therapy (hormone therapy)
Colorectal cancer^71^	Surgery	12	**Oncological outcomes:** Long-term survival, cancer recurrence and resection margins **Operative outcomes:** Anastomotic leak, perioperative survival, surgical site infection, stoma rates and complications and conversion to open operation (where appropriate) **Quality of life:** Physical function, sexual function, faecal incontinence and faecal urgency

### Weight-management intervention design

The design of weight-management intervention (in terms of dose and duration) needs to be driven by practicalities as well as the desired magnitude of change in body composition (e.g., body fatness and skeletal muscle mass)—this approach has the greatest likelihood of positively influencing patient and clinical outcomes.^58^ Caloric intake is the cornerstone of weight loss, but regular physical activity and structured exercise programmes have important roles to play in all aspects of weight management.^59^ Importantly, physical activity and exercise can preserve skeletal muscle mass during dietary-induced fat loss,^60,61^ thereby helping to protect against the adverse impact of sarcopenia on cancer-survival outcomes^31,62^ and increasing total daily energy output.^63^ Physical activity post diagnosis is associated with improved survival outcomes for patients with breast, colorectal or prostate cancer.^64^ Furthermore, an international consensus statement concluded that sufficient evidence now exists to show that regular exercise improves several cancer-related health outcomes, including anxiety, depressive symptoms, fatigue, physical functioning and health-related quality of life in cancer survivors.^65^

The growing body of effective weight-loss programmes (BRRIDE,^66^ DIRECT^67^ and DPP^68^) that have achieved clinically relevant changes (e.g., diabetes remission) in cancer and non-cancer patients provides a good starting point for intervention design. However, translating these programmes into cancer-survivorship populations might require significant patient involvement to ensure that the components (notably, dietary and structured exercise or physical activity goals) can be achieved by those with a wide range of abilities, disabilities, emotional needs, available time and financial circumstances. Furthermore, insights from behavioural science^69^ provide guidance for embedding strategies to support long-term behavioural change, which are anchored in robust psychological theory and evidence-based behaviour-change techniques. The potential of remote support offered by digital and other ‘smart’ technologies (in particular, to people with co-morbid conditions such as cognitive and sight impairments) provides further scope to engage with vulnerable people, including those living in rural communities.

### Feasibility studies

**Fig. 2:**
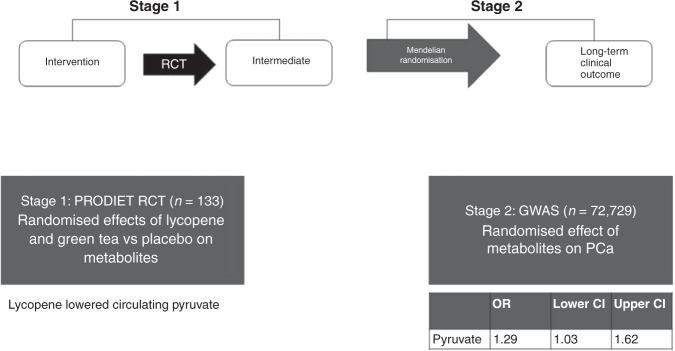
Two-step Mendelian Randomisation procedure: integration of feasibility randomised controlled trial (RCT) results with MR to predict the long-term effect of interventions. Introduction to Mendelian randomisation: Mendelian randomisation is a form of instrumental variable analysis that uses genetic variants as instruments to examine the causal effects of modifiable exposures on outcomes of interest. This method depends on the existence of genetic variants that are robustly associated with metabolite levels. In the example outlined here, the results of a feasibility RCT of dietary interventions for the prevention of prostate cancer were carried forward to a large-scale Mendelian randomisation analysis to infer the causal effect of the interventions on prostate cancer risk via intermediate metabolites. Step 1 assessed the randomised effects of lycopene and green-tea consumption for 6 months versus placebo on 159 serum metabolic traits, quantified by nuclear magnetic resonance (NMR), amongst 133 men enrolled in the ProDiet randomised controlled trial. Step 2 used Mendelian randomisation to assess the effects of those metabolic traits altered by the intervention on prostate cancer risk, using genome-wide association studies (GWAS) summary statistics from the Prostate Cancer Association Group to Investigate Cancer Associated Alterations in the Genome (PRACTICAL) consortium. The lycopene intervention lowered circulating levels of pyruvate, a change that the Mendelian randomisation analysis suggested was associated with decreases in prostate cancer risk (a genetically instrumented SD increase in pyruvate increased the odds of prostate cancer by 1.29 (1.03, 1.62, *P* = 0.027)). Lycopene lowered the levels of pyruvate, which our Mendelian randomisation analysis suggests may be causally related to reduced prostate cancer risk. By combining the results of a feasibility study with Mendelian randomisation, it has been possible to identify potential intermediate mechanisms through which interventions might be influencing cancer risk (see 767,68 (step 2)).

Finally, feasibility trials are an essential starting point for definitive randomised controlled trials with respect to gauging patient acceptability and tolerability, and gleaning valuable qualitative and quantitative data about recruitment, implementation, retention and indicative effects. One novel method that could transform the interpretation of feasibility trials is the use of Mendelian randomisation. In this context, feasibility studies can estimate the intervention effects on intermediate endpoints that might be on the causal pathway to clinical outcomes. Using a two-step process, the results of small-scale feasibility studies can be used to inform much larger-scale two-sample Mendelian randomisation studies. This approach could provide novel insight into the causal effects of an intervention on important intermediate endpoints and possible long-term clinical endpoints (see Fig. 2). In this way, Mendelian randomisation can then be used alongside feasibility studies to optimise intervention development and delivery, including more accurate outcome predictions for fully powered conventional randomised controlled trials,^70^ as outlined in Fig. 2.^71^

## Conclusions

It is timely to extend our knowledge of weight management by moving from epidemiology studies to interventional research, as it relates to EBF in the context of cancer treatment and survivorship. This increased knowledge will improve our understanding of the health benefits to be gained from optimising body composition in people living with and beyond common cancers, who constitute a significant health burden worldwide. Interventions need to be complex but pragmatic in design, while encompassing multidisciplinary methodological approaches aimed at improving our understanding of causal mechanisms. These endeavours are urgently needed to develop evidence-based strategies for mitigating the adverse impact of EBF in a growing global population of cancer survivors^67^ living in increasingly obesogenic societies.

## Data Availability

Not applicable.
